# Excellent age hardenability with the controllable microstructure of AXW100 magnesium sheet alloy

**DOI:** 10.1038/s41598-020-79390-z

**Published:** 2020-12-29

**Authors:** Sumi Jo, Lawrence Whitmore, Sangkyu Woo, Ainhoa Urrutia Aramburu, Dietmar Letzig, Sangbong Yi

**Affiliations:** 1 Magnesium Innovation Centre (MagIC), Helmholtz-Zentrum Geesthacht, Max-Planck-Str. 1, 21502 Geesthacht, Germany; 2 Department of Chemistry and Physics of Materials, Salzburg University, Jakob-Haringer-Str. 2a, 5020 Salzburg, Austria; 3 Department of Mechanical Engineering, Mondragon University, Loramendi Kalea 4, 20500 Arrasate, Spain

**Keywords:** Materials science, Physics

## Abstract

Age-hardenability and corresponding improvement of the mechanical properties of Mg–1Al–0.7Ca and Mg–1Al–0.7Ca–0.7Y alloy sheets are addressed with respect to the microstructure and texture evolution during thermomechanical treatments. A fine grain structure and weak texture with the basal pole split into the sheet transverse direction are retained in the Mg–1Al–0.7Ca–0.7Y sheet even after the homogenization at 500 °C, due to the grain boundary pinning by Y-containing precipitates possessing a high thermal stability. Contrarily, the Mg–1Al–0.7Ca sheet shows a coarse microstructure and basal-type texture after the homogenization. The peak-aged condition is attained after the aging at 250 °C for 1800 s of both homogenized sheets, while the Y-containing sheet shows a higher hardness than the Mg–1Al–0.7Ca sheet. TEM analysis and thermodynamic calculation show the formation of metastable precipitates composed of Al, Ca, Y and Mg in the Mg–1Al–0.7Ca–0.7Y sheet at the homogenized and peak-aged conditions. A significant increase in the yield strength is obtained in the peak-aged condition from 162 MPa after the homogenization to 244 MPa, which arises from the increased size and number density of the precipitates. The high age-hardenability of the Mg–1Al–0.7Ca–0.7Y sheet attributes to the superior mechanical properties with an improved ductility promoted by the weak texture.

## Introduction

Mg has a limited number of deformation modes activating at low temperature, and the formability of Mg sheet is determined depending on the grain orientations and their distribution; namely, crystallographic texture. Based on the texture control the formability of Mg sheet has been significantly improved in the last decade, and several Mg alloy sheets exhibit formability comparable to the 6000 series Al alloy sheet at room temperature^1,2^. Highly formable Mg sheet alloys have been developed by altering the basal-type texture to diversely distributed grain orientations^3–7^, which is attributed to the higher activation of non-basal deformation modes and restricted grain boundary motion during recrystallization annealing^8–10^. It is well acknowledged that the texture weakening can be achieved by alloying the rare earth elements (RE) or Ca with simultaneous addition of Zn. Meanwhile, texture weakening brings out to increase the sheet formability by scarifying strength instead, that is known as the dilemma between strength and formability^11^. Although several studies reported the great ductility or formability, such as the Erichsen index (I.E.) higher than 8.0 mm or fracture strain close to 30% at room temperature, in various Mg alloys containing Ca or RE the yield strength (YS) of the sheets is lower than 150 MPa^12–14^. Recently, a number of studies have been carried out to improve the YS and formability of Mg sheet at the same time^1,15–19^. The improved YS of the developed Mg sheets are laid within the range between 200^17,19^ to 238 MPa^1^. Trang et al.^18^ reported the AZMX3110 alloy sheet with high strength, 219 MPa of YS and high formability, 8 mm of I.E.. The segregated Ca and Zn elements at grain boundaries of the AZMX3110 sheet suppress its movement during recrystallization, and contribute to the formation of rather weak texture. The texture weakening is promoted by increasing the formation of Al–Mn primary particles and leaving more Ca as solutes via twin-roll casting and a higher Mn addition into the AZX310 alloy^18^. In addition, age-hardening is an effective way to improve the sheet strength when it achieves a sufficiently high peak-hardness in a short aging time, namely, high aging response. Bian et al.^1^ reported, in this regard, that the Ca and Zn solutes segregate into basal <a> dislocations, and thus effectively hinder dislocation motion, resulting in bake-hardening of AZXM1110 alloy with 7.8 mm of I.E. in T4 condition and 238 MPa of YS at the peak-aged condition. Ca has become a key element for age-hardening, since its high affinity to other elements, such as Al and Zn^20^. Indeed, a high age-hardenability was reported in the Ca-containing Mg–Al based^21–24^ and Mg–Zn based alloys^25–28^. Likewise, Y is also well acknowledged as an effective element for improving formability of Mg alloys by texture weakening in correlation with the change in stacking fault energy^29^, and for improved strength via age-hardening^30,31^. In this regard, the role of Ca and Y on the age hardenability and texture evolution is examined by performing a comparative study of AX10 sheet from its Y-containing counterpart AXW100 alloy. The microstructural features of the both alloys were investigated by using a transmission electron microscopy (TEM) including high-resolution TEM and energy-dispersive X-ray spectroscopy (EDS), and thermodynamic calculation.

## Results and discussion

**Figure 1:**
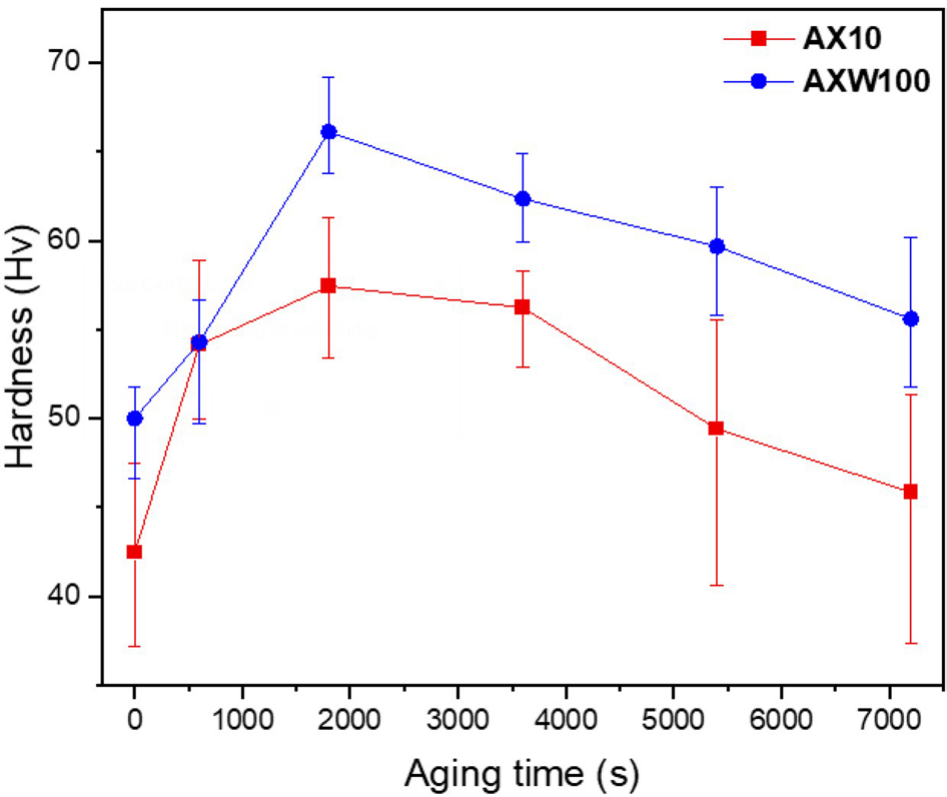
Hardness of the investigated AX10 and AXW100 alloy sheets according to the isothermal aging treatment at 250 °C.

**Figure 2:**
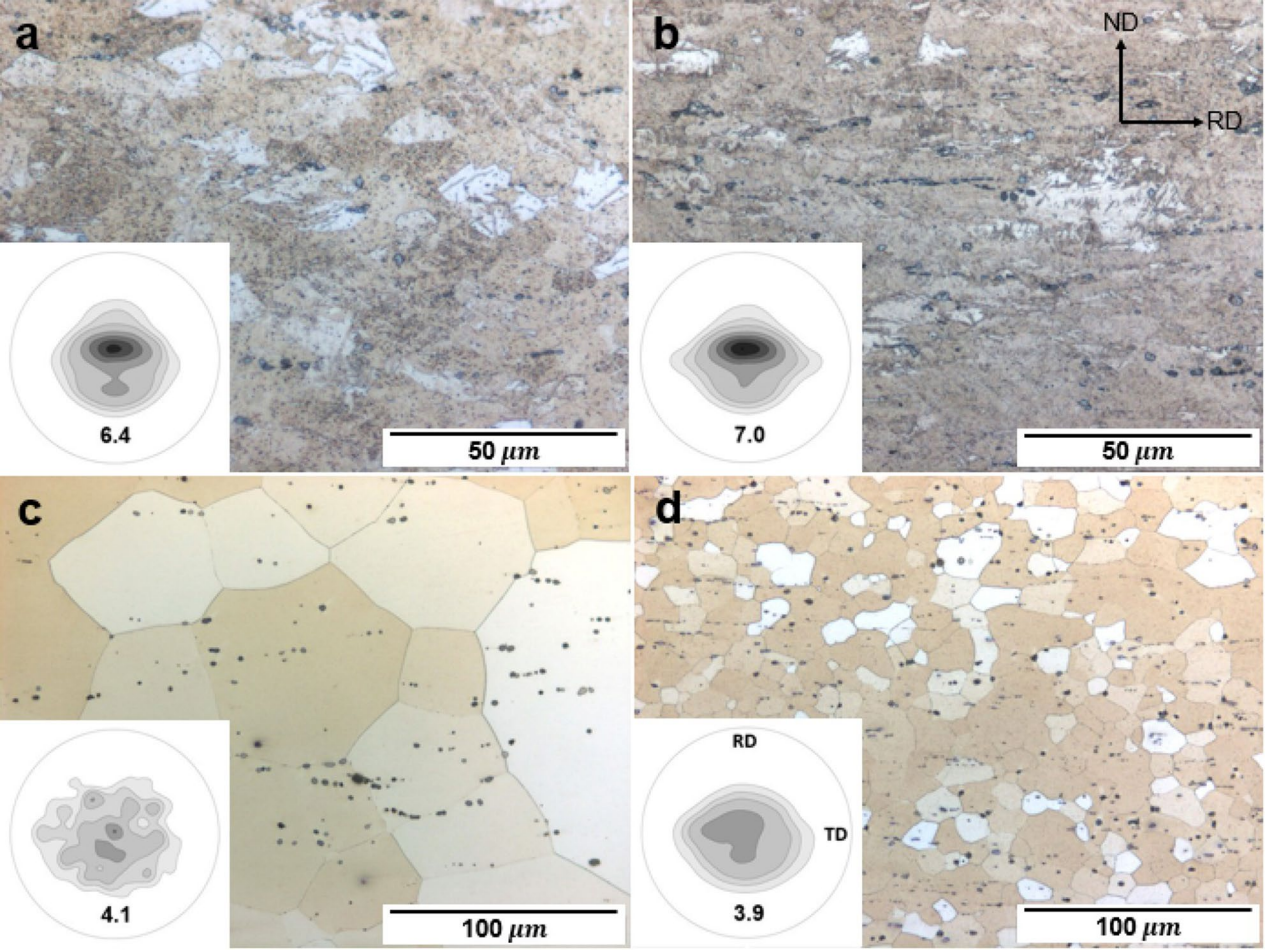
Optical micrographs and corresponding (0002) pole figures of (**a**,**b**) as-rolled and (**c**,**d**) homogenized AX10 and AXW100 alloys, respectively. Maximum pole densities of the (0002) pole density = 6.4, 7.0, 4.1 and 3.9 m.r.d. (multiple of random distribution), respectively.

Hardness evolution during the aging treatment at 250 °C of the homogenized AX10 and AXW100 sheets is plotted in Fig. 1. The time to reach the peak-hardness is 1800 s for both AX10 and AXW100 sheets, and peak-hardness is 57 HV and 66 HV, respectively. The hardness value of the AXW100 sheet are higher than that of the AX10 sheet during the entire aging time as well as the homogenized state. The optical micrographs and the recalculated (0002) pole figures of the as-rolled and homogenized sheets are shown in Fig. 2. The as-rolled sheets of AX10 and AXW100 alloys have similar microstructure with twins, deformation bands and secondary phases (Fig. 2a,b). The secondary phases aligned along the rolling direction (RD) are Mg_2_Ca phase in AX10, and Mg_2_Ca and Mg_2_Y phases in AXW100, which were not dissolved during the homogenization treatment of the cast materials. The thermal stability of these phases are also shown from the thermodynamic calculation, which indicates the corresponding phases are formed as primary phase at the equilibrium conditions (Fig. 7).

On the other hand, the homogenized sheets show a distinct difference in the average grain sizes between the AX10 and AXW100 alloys, 65 $\upmu\text{m}$ and 13 $\upmu\text{m}$, respectively. Grain coarsening occurs during the homogenization of the AX10 alloy, whereas the AXW100 alloy maintains the grain size almost similar to the as-rolled condition.

The recalculated (0002) pole figure of each sheet is presented in the inset into the corresponding optical micrograph. The as-rolled AX10 sheet shows the basal poles tilted towards the RD, while the AXW100 sheet additionally forms a basal pole spread along the transverse direction (TD). The development of the texture with basal pole spread is considered as a typical feature of RE-containing Mg alloys^32–34^. The texture of the both sheets after the homogenization annealing show clear differences while the maximum pole densities of the (0002) pole figures are similar, P = 4.1 and 3.9 in AX10 and AXW100, respectively. The homogenized AX10 sheet shows a spotted distribution of basal texture, which is derived from the coarse microstructure as shown in Fig. 2c. The AX10 sheet shows a basal-type texture in which most basal pole is nearly parallel to the sheet normal direction (ND). The AXW100 sheet shows a weak texture with the basal poles evenly tilted from the ND towards the RD and TD. As many studies on sheet formability of Mg alloys have shown, especially in Mg–Zn–RE and Mg–Zn–Ca alloys^4,7,8,35,36^, such weak texture with the tilted basal pole to the TD formed in the AXW100 sheet is to be beneficial for low temperature formability. The textures of the homogenized sheets are maintained during the aging treatment, as can be expected from the relatively low aging temperature and no visible change in the grain structure.

**Figure 3:**
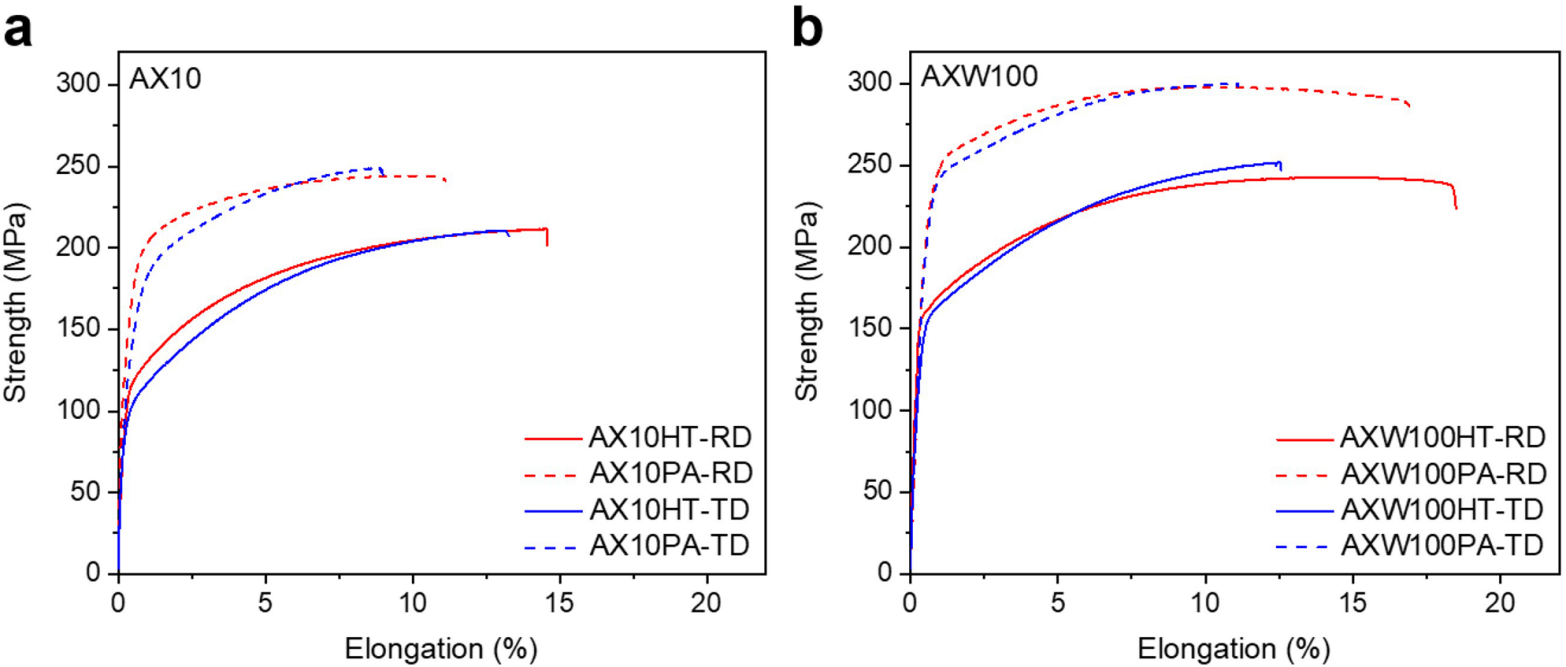
Tensile properties of the homogenized and peak-aged (**a**) AX10 sheets and (**b**) AXW100 sheets along the RD and TD at room temperature. Red and blue curves indicates the curves of the investigated alloy sheets along the RD and TD, respectively. The solid and dotted lines present the curves of the homogenized and peak-aged alloy sheets, respectively. HT indicates the homogenized samples heat-treated at 500 °C for 3 h. PA indicates the peak-aged samples heat-treated at 250 °C for 1800 s following the homogenization treatment.

The tensile behaviors of the AX10 and AXW100 alloy sheets are plotted in Fig. 3a,b, respectively, and summarized in Table 1. The YS of both alloy sheets are remarkably improved through the aging treatment along both RD and TD. The YS of the AX10 increases by aging treatment at 250 °C for 1800 s, which corresponds to the peak-aged condition, from 118 and 103 MPa to 169 and 151 MPa in the RD and TD, respectively. The lower YS of the homogenized sheet along the TD than that along the RD can be understood by the texture of which the basal pole broadening toward the TD as shown in Fig. 2c. The AXW100 alloy sheet shows also a significant increase of the YS after the aging treatment, from 162 and 158 MPa to 244 and 239 MPa in the RD and TD, respectively. The yield anisotropy of the homogenized AXW100 is lower than that of the AX10 alloy sheet, which results from the relatively uniformly distributed basal poles in the AXW100 sheet, as presented in the Fig. 2d. The AXW100 sheet shows more significant improvement of YS by the aging treatment as well as the higher fracture strain than the AX10 at the homogenized and peak-aged condition along the RD, 18% and 17%, respectively. The fracture strain of the peak-aged AXW100 is almost identical to that of the homogenized AXW100 sheet. That is, no sacrifice of ductility by age-hardening of the AXW100 sheet. Contrarily, the fracture strain of the homogenized and peak-aged AX10 along the RD is 15% and 11%, respectively, indicating the decreased ductility by the aging treatment. The homogenized AXW100 alloy shows not only the higher tensile properties and hardness value, but also the weaker texture with tilted basal poles towards the RD and TD in comparison to the AX10 alloy. The fine grain structure of the homogenized AXW100 alloy is responsible to these improved mechanical properties and weaker texture.

**Table 1:** Grain sizes (GS) and yield strength (YS), ultimate tensile strength (UTS) and elongation (El) of the homogenized and peak-aged AX10 and AXW100 sheets along the RD and TD.

Samples	GS ($\upmu\text{m}$)	YS (MPa)		UTS (MPa)		El (%)	
		RD	TD	RD	TD	RD	TD
**AX10**							
Homogenized	65	118	103	212	210	15	13
Peak-aged	–	169	151	244	249	11	9
**AXW100**							
Homogenized	13	162	158	243	251	18	13
Peak-aged	–	244	239	298	299	17	11

**Figure 4:**
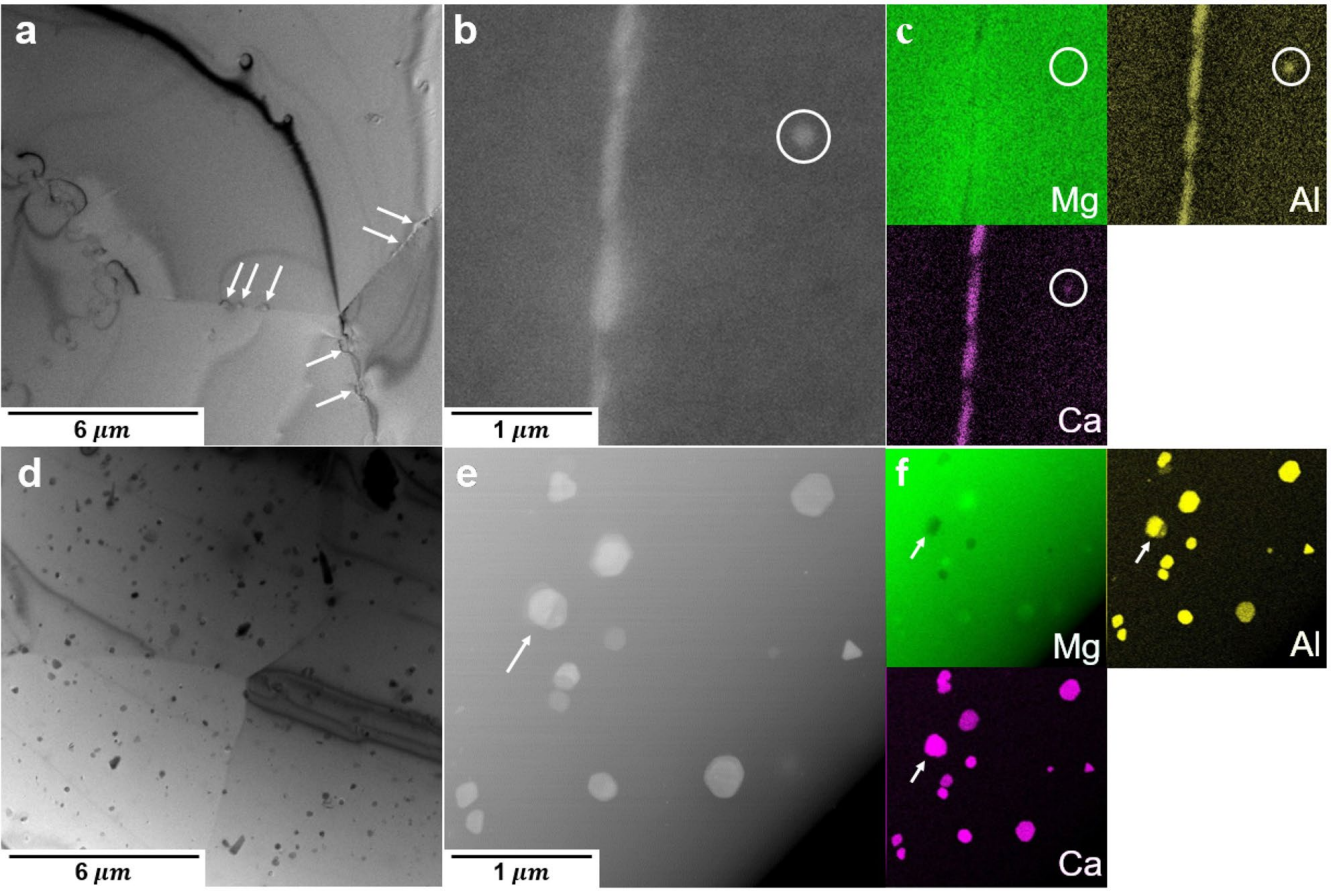
TEM images of (**a**) the homogenized and (**d**) peak-aged AX10 alloy sheets. High-magnified STEM images and corresponding EDS maps of (**b**,**c**) the homogenized and (**e**,**f**) peak-aged AX10 alloy sheets for Mg, Al and Ca. The arrows in (**a**) indicate the contrast in vicinity of grain boundaries. The circles in (**b**,**c**) correspond to a C36 particle consisted of Mg, Al and Ca and the arrow in (**e**,**f**) indicates an inhomogeneous distribution of alloying elements in a particle.

The characteristic differences in the microstructure evolution between the AX10 and AXW100 sheets during the aging treatment were investigated by using electron microscopy. TEM micrographs and EDS maps of the homogenized and peak-aged AX10 alloy are shown in Fig. 4a–f, respectively. The homogenized AX10 sheet shows clear diffraction contrast at the grain boundaries to the matrix as indicated by white arrows in Fig. 4a, which implies either intensive segregation of alloying elements or the presence of precipitates. The STEM image in the vicinity of grain boundary and the corresponding EDS maps for Mg, Al and Ca are illustrated in Fig. 4b,c. The segregation of Al and Ca is found along the grain boundary of the homogenized AX10 sheet. According to the previous studies^8,18^, the boundary segregation of Ca and Zn in an ordered manner is energetically more stable and would hamper the grain boundary motion and, consequently, lead the texture weakening. The present results, however, indicate that the co-segregation of Al and Ca at grain boundaries inadequately hinders the boundary motion, such that the coarse grain structure and basal-type texture were formed in the homogenized AX10 (Fig. 2c).

The AX10 sheet at the peak-aged condition shows the round precipitates in the matrix and at the grain boundaries, which obviously leads to the age-hardening. Figure S2 in the supplementary material shows the microstructures of AX10 sheet before and after the aging treatment, which clearly indicates the microstructural difference caused by the aging treatment. In addition, Fig. S2b demonstrates the difference in size and number density between the newly formed precipitates during the aging and the secondary phases existing from the solidification process. The average size and number density of the precipitates in the peak-aged AX10 sheet are 174.3 nm and $1.4\times 10^{12}$ m^−2^ (Table 2). Precipitates formed at the peak-aged condition of the AX10 sheet are divided into two different phases. One consists of Mg, Al and Ca, and the other mainly of Al and Ca. Equilibrium phase fraction of the AX10 alloy from the thermodynamic calculation using Pandat software^37^, Fig. 7a indicates the formation of Al_2_Ca (C15) phases at the aging temperature. The precipitate found at the homogenized condition, marked with a white circle in Fig. 4b, consists of Mg, Al and Ca, indicating that the particle is (Mg,Al)_2_Ca (C36) phase. According to Raghavan^38^ and Suzuki et al.^39^, C36 phase exists as an equilibrium phase and has an intermediate structure between Mg_2_Ca (C14) and C15 phases. Based on the structural similarity between C36 and C14 phases, it is plausible that C36 phase could exist with C14 phase during the homogenization treatment. Moreover, the C36 phase contributes also to the age-hardening. The EDS maps of the peak-aged AX10 sheet, Fig. 4e,f show the presence of both C36 and C15 phases. Interestingly, some precipitates have an inhomogeneous distribution of Mg and Al, such as the precipitate indicated with the white arrow in Fig. 4e. That is, one part of the particle seemed to be C36, and the other is C15 phase. This finding indicates that the C15 precipitates at the aged condition are either newly formed or transformed from C36 phases which exist at the homogenized condition, during the aging treatment at 250 °C.

**Table 2:** Particle size and number density of the precipitates in the homogenized and peak-aged AX10 and AXW100 sheets, respectively.

Samples	Particle size ($\mathrm{nm}$)	Number density (${\text{m}}^{-2})$
**AX10**		
Homogenized	–	–
Peak-aged	174.3	$1.4\times 10^{12}$
**AXW100**		
Homogenized	30.5 / 84.0	${18.7}\times {10}^{12}$
Peak-aged	59.6 / 100.7	${39}{.}{4}\times {10}^{12}$

**Figure 5:**
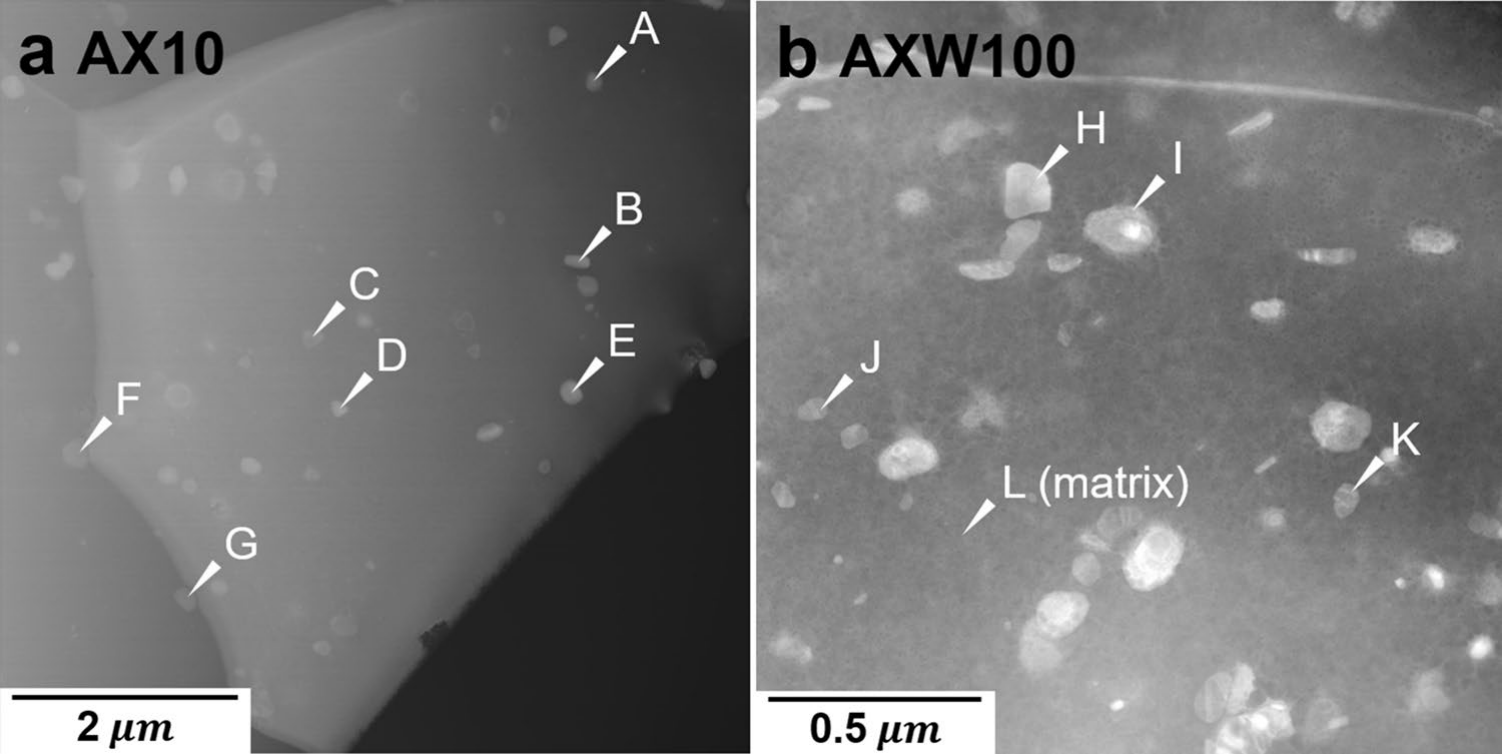
STEM images of the peak-aged (**a**) AX10 and (**b**) AXW100 sheet. The EDS results on the precipitates marked by arrows A to G in (**a**) and H to L in (**b**) are given in Tables 3 and 4, respectively.

The STEM image of the peak-aged AX10 sheet is demonstrated in Fig. 5a, and the EDS analysis was conducted on the precipitates marked with white arrows. The chemical compositions and the stoichiometry of Al and Ca of the marked precipitates are listed in Table 3. All analyzed precipitates consisted of Al, Ca and Mg. The amount of Mg are excluded due to the uncertainty of the measurement, because the matrix around the analyzed precipitates is included in the interaction volume of electrons. The point A, B, C and E presents the stoichiometry of Al and Ca is almost 2:1, indicating C15 phase. Similarly, the D, F and G with the stoichiometry of nearly 1:1, match to C36 phase. The above results indicate that the age-hardening in the AX10 sheet is contributed to the formation of C15 and C36 phases.

**Table 3:** Chemical composition (in at.%) of the precipitates in the peak-aged AX10 sheet indicated by white arrows in Fig. 5a, analyzed by STEM-EDS.

Point	Elements			Stoichiometry
	Al	Ca	Mg	Al:Ca
A	14.72	5.99	79.28	5:2
B	40.03	17.33	42.64	40:17
C	11.20	5.12	83.68	11:5
D	11.89	10.04	78.07	6:5
E	33.89	20.01	46.10	17:10
F	8.86	11.58	79.56	3:4
G	15.24	14.90	69.86	1:1

**Figure 6:**
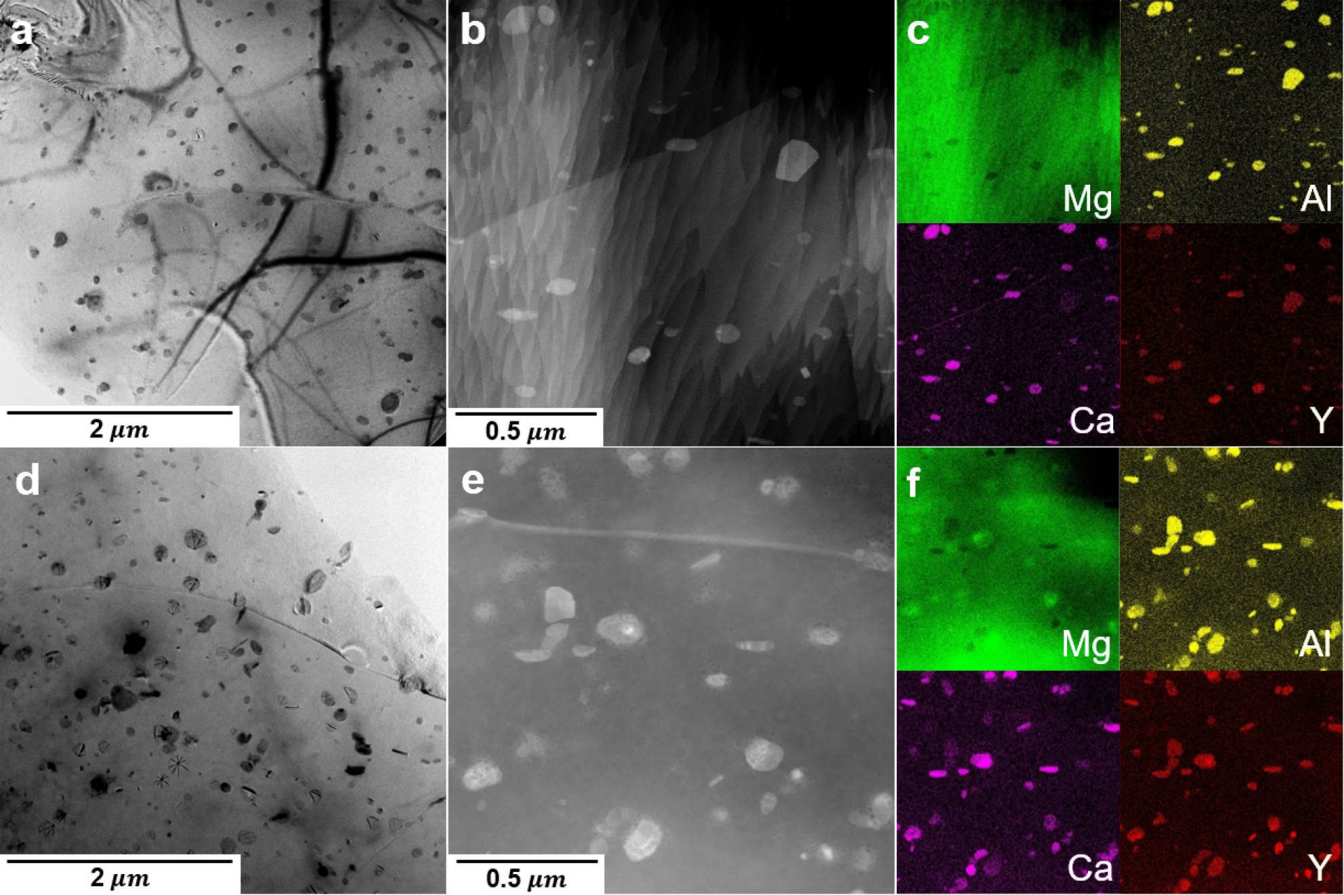
TEM images of (**a**) the homogenized and (**d**) peak-aged AXW100 alloy. High-magnified STEM images and corresponding EDS maps of (**b**,**c**) the homogenized and (**e**,**f**) peak-aged AXW100 alloy for Mg, Al, Ca and Y.

Figure 6 demonstrates the microstructure and corresponding EDS maps of the homogenized and peak-aged AXW100 alloys. The number density of precipitates of the homogenized and peak-aged AXW100 alloys are ${18.7}\times {10}^{12}{\,{\text{m}}}^{-2}$ and ${39.4}\times {10}^{12}\,{\text{m}}^{-2}$ (Table 2). The number density of the precipitates increases more than double after the aging treatment at 250 °C for 1800 s. The precipitates show a bimodal distribution in size and, therefore, are divided into two groups based on the average size, 30.5 nm and 84.0 nm after the homogenization and 59.6 nm and 100.7 nm at the peak-aged condition. These results indicate that the precipitates at the homogenized condition grow during the aging treatment in addition to the formation of more precipitates. That is, the increase in the number density with the simultaneous growth of the precipitates during the aging treatment significantly improves the hardness and YS of the AXW100 sheet (Fig. 3), according to the Orowan mechanism. The shorter distance between the precipitates and the larger size of the precipitates require higher stress for dislocations to pass through the precipitates, i.e. increase in the YS^40^.

It is to mention that the homogenized AXW100 shows a slightly lower YS and, accordingly, a lower hardness value than that of the peak-aged AX10 sheet, 162 MPa and 169 MPa, respectively, even though a higher number density of the precipitates is determined in the former. To understand this interesting behavior, various factors that influence the mechanical properties are to be considered, in addition to the strengthening by the precipitation. The effect of Y addition on the texture weakening and concurrent ductility increase is well acknowledged as a result of enhanced activation of non-basal deformation modes^32^ and reduced stacking fault energy^29^, and the corresponding results are found in the present study (Figs. 2b, 3b). In this context, the Y addition can contribute to a relatively lower strength of the homogenized AXW100 sheet. Another factor which influences the mechanical strength is the interdistance and the size of precipitates. In the present study, the precipitates size in the homogenized AXW100 sheet varies from 18 to 130 nm and the average size of the lower 50% of all measured precipitates is 30 nm, and those in the peak-aged AX10 sheet are 62–293 nm and 87 nm. It can be, therefore, considered that the fine precipitates in the homogenized AXW100 sheet possess a relatively weaker ability to impede the dislocations motion due to the small size, in comparison to the peak-aged AX10 sheet. Similar cases were reported by Hidalgo-Manrique et al.^41^ that the MN11 extrudate showed the highest YS when the precipitates length became 150 nm through the annealing treatment. Robson and Paa-Rai^42^ reported that the hardness of the Mg–6 wt% Zn alloy could be improved in accordance with the increase of precipitates size from 50 to 250 nm by the aging treatment at 200 °C.

**Figure 7:**
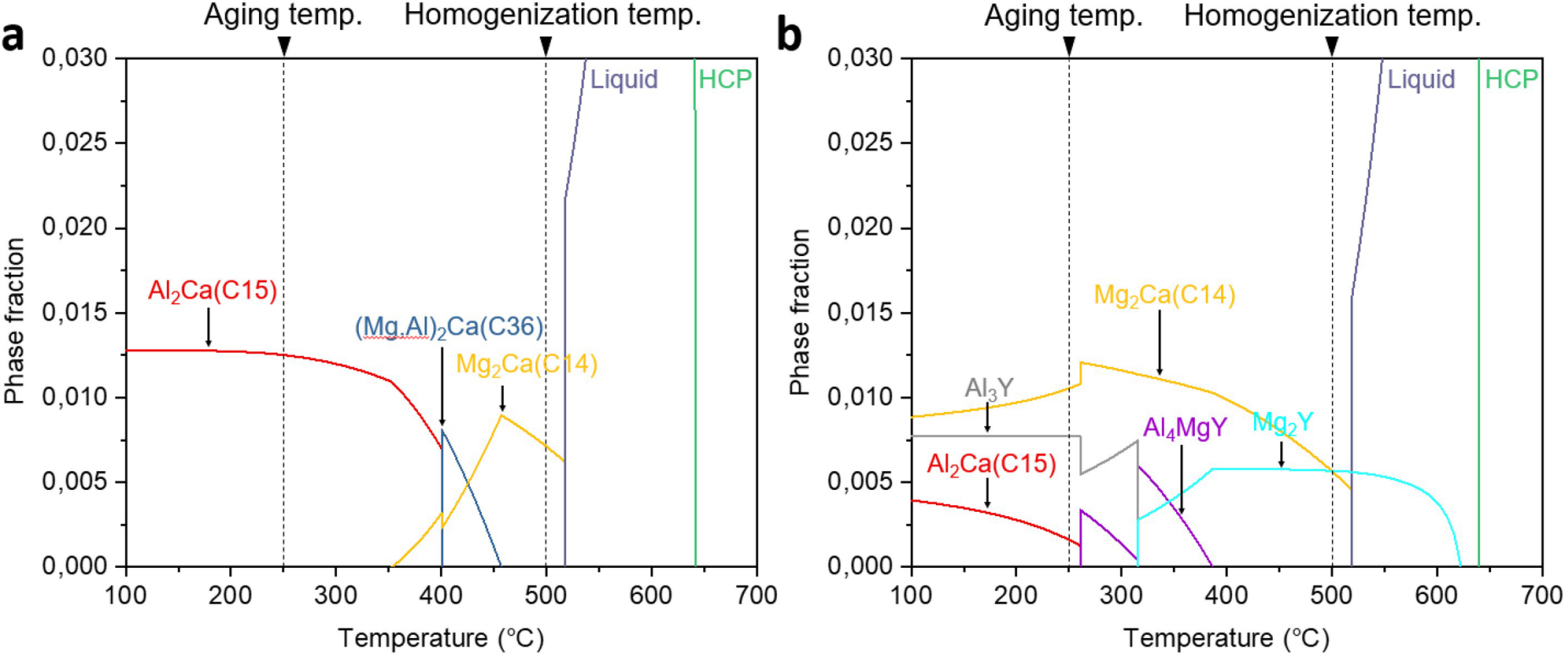
Equilibrium phase fraction of the AX10 and AXW100 alloys calculated using thermodynamic calculation software Pandat^37^.

The STEM image and chemical compositions of the precipitates formed in the peak-aged AXW100 alloy are shown in Fig. 5b and Table 4. The EDS analysis indicates that the precipitates consist of Al–Ca–Y or Al–Ca–Y–Mg, which are not found in the thermodynamic calculation of the equilibrium condition (Fig. 7b). The stoichiometry among Al, Ca and Y of the analyzed precipitates, listed in Table 4, is ambiguous in comparison to the equilibrium phases, i.e. Al_2_Ca, Mg_2_Ca and Al_3_Y, at the aging temperature of 250 °C calculated by thermodynamic calculation.

**Table 4:** Chemical composition (in at.%) of the precipitates in the peak-aged AXW100 sheet indicated by white arrows in Fig. 5b, analyzed by STEM-EDS.

Points	Elements				Stoichiometry
	Al	Ca	Y	Mg	Al:Ca:Y
H	9.84	0.45	1.73	87.98	21:1:4
I	12.95	5.28	2.40	79.37	27:11:5
J	4.62	1.82	1.24	92.33	19:7:5
K	11.04	2.53	4.99	81.45	4:1:2
L	0.24	0.05	0.03	99.68	24:5:3

**Figure 8:**
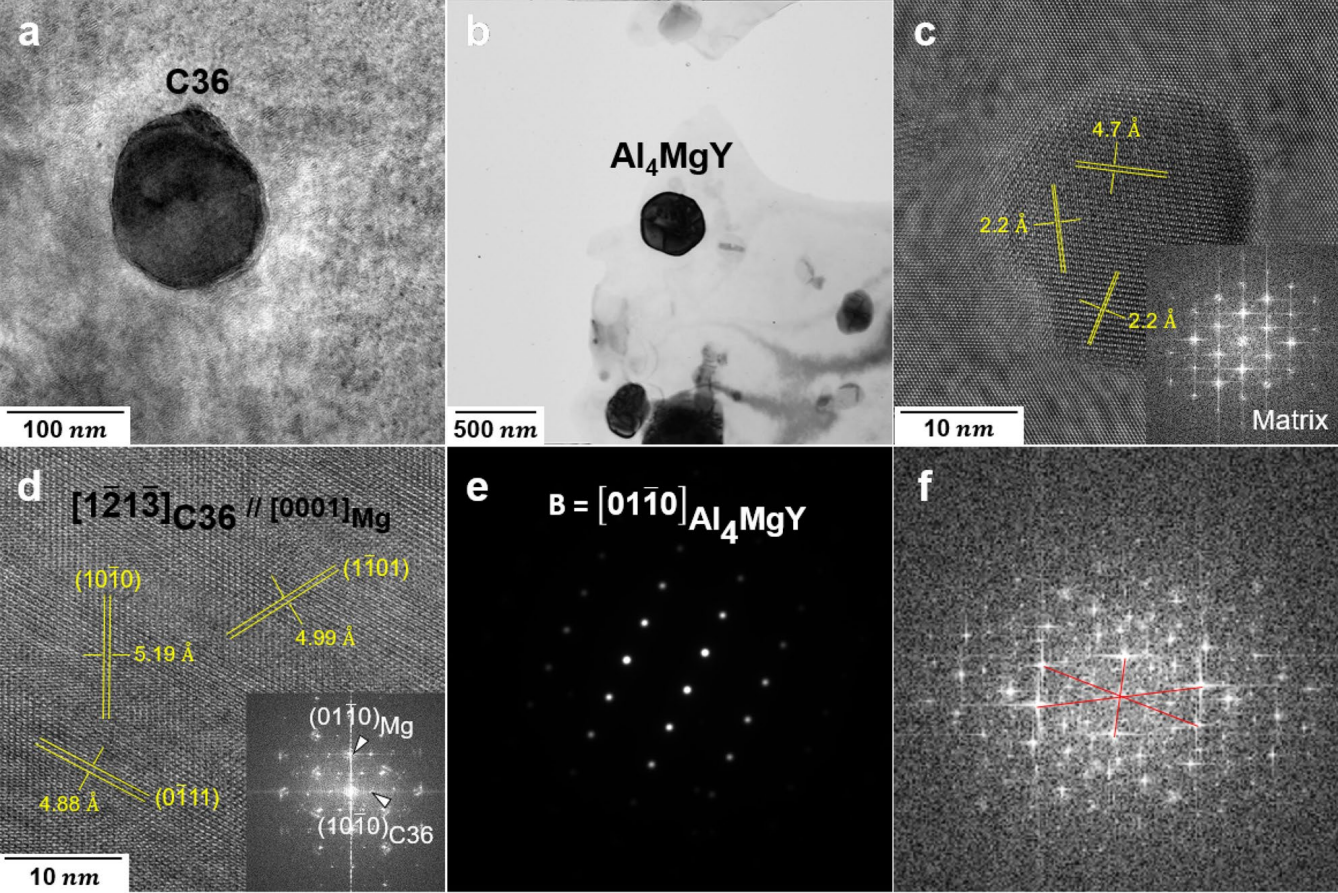
TEM images showing the several precipitates in the homogenized AX10, AXW100 and peak-aged AX10 alloy sheets. (**a**,**d**) HRTEM image and FFT pattern along ${[0001]}_{\text{Mg}}$ axis of C36 particle in the homogenized AX10 sheet, (**b**,**e**) TEM bright field image and SADP of Al_4_MgY particle in the homogenized AXW100 sheet, and (**c**,**f**) HRTEM image and FFT pattern of the particle in the peak-aged AX10 sheet.

TEM images and corresponding HRTEM and selected area diffraction pattern (SADP) of a precipitate in the homogenized AX10 and AXW100 alloys are presented in Fig. 8a,b,d,e, respectively. The HRTEM and FFT pattern from the precipitate shown in Fig. 8a, are indexed as C36 in a hexagonal structure with the lattice parameter^39^ of a = 5.96 Å and c = 19.79 Å, and the orientation relationship (OR) to the Mg matrix is ${[0001]}_{\text{Mg}}$//${\left[{1}\bar{2}{\text{1}}\bar{3}\right]}_{\text{C36}}$, ${\text{(01}\bar{1}\text{0)}}_{\text{Mg}}$//${\text{(2}\bar{1}\bar{1}\text{1)}}_{\text{C36}}$. The SADP of the particle in the homogenized AXW100 alloy was identified as Al_4_MgY phase in a hexagonal structure with the lattice parameter^43^ of a = 5.33 Å and c = 8.57 Å and OR is ${[0001]}_{\text{Mg}}$// ${\text{[01}\bar{1}\text{0]}}_{{\text{Al}}_{4}{\text{MgY}}}$.

On the other hand, the HRTEM and corresponding FFT pattern of a particle in the peak-aged AX10 alloy, Figure 8c,f show the interplanar distances are 4.7 Å, 2.2 Å, and 2.2 Å and the interplanar angles between the planes are 76°, 30° and 75°, respectively. These values do not precisely match to the phases expected to be formed in the Mg–Al–Ca alloy system such as C14, C15 and C36 phases. The particle with size of 20 nm in Fig. 8c is much finer than that of the average size. Since the particle have insufficient size to take a stable structure, it might have a metastable structure, which are not be indexed. Likewise, the chemical composition of the particles in the homogenized and peak-aged AXW100 alloy sheets show the unclear matching results. The particles in the homogenized and peak-aged AXW100 alloy are composed of Al–Ca–Y and Al–Ca–Y–Mg deviate from the equilibrium composition. The size of the fine precipitates, especially newly formed precipitates in size range of 20–35 nm in the homogenized AXW100 alloy sheet are similar to that of the particle in Fig. 8c, and, therefore, it is considered these particles have the metastable structure.

A diffusion coefficient of Y solute in Mg is approximately 100 times slower than that of Ca solute at 227 °C (500 K)^44^. The slow diffusion rate of Y solute contributes to the high thermal stability of the Y-containing phases, e.g. Al_4_MgY, to make difficult to dissolve during the homogenization treatment. In addition, the mixing enthalpy of Al–Ca, Al–Y and Mg–Y are − 20 kJ/mol, − 38 kJ/mol and − 6 kJ/mol, respectively^20^. Due to the lowest mixing enthalpy of Al–Y, it can be also supposed that the precipitation of Al containing phase accompany with the segregation of Y solutes. These factors, i.e. low diffusion coefficient and high mixing affinity of Y to the other elements, additionally support the formation of the metastable phases containing Y deviating from the equilibrium composition in the AXW100 alloy.

The equilibrium compositions of the matrix phase at the homogenization and aging temperatures were calculated for the AX10 and AXW100 alloys (Table 5). The results show that the amount of Al and Ca solutes dissolved in the matrix significantly decrease at the aging temperature. In other words, Al and Ca solutes mainly contribute to bring out the precipitation behavior at the aging temperature, i.e., formation of new precipitates and simultaneous thickening of the pre-existed metastable phases. As a result, the metastable phases in the homogenized and peak-aged AXW100 alloy sheet are formed by the collaborative behavior of Al, Ca and Y solutes, and they contribute to the improved aging response of the AXW100 alloy sheet.

**Table 5:** Calculated composition (in at.%) of the matrix for the AX10 and AXW100 alloys at equilibrium. The amount of Al, Ca and Y solutes dissolved in the matrix at the homogenization (500 °C) and aging (250 °C) temperature.

Samples	Elements		
	Al	Ca	Y
**AX10**			
Homogenized	0.76	0.19	–
Peak-aged	0.11	0.0091	–
**AXW100**			
Homogenized	0.46	0.24	$3.85 \times 10^{-3}$
Peak-aged	0.069	0.023	$1.94 \times 10^{-6}$

According to the thermodynamic calculation in Fig. 7, it is clearly shown that the accumulated fraction of the secondary phases of the AXW100 alloy at the homogenization temperature is almost twice that of the AX10 alloy. It corresponds to the larger number density of the precipitates in the homogenized AXW100 alloy, including the Y-containing fine precipitates formed at the grain boundaries, than that in the homogenized AX10 alloy, as shown in Figs. 4a and 6a. It is obvious that those fine precipitates play an important role to the grain boundaries pinning such that the relatively fine grain structure is maintained during the homogenization at 500 °C. Further, the fine precipitates play a role to weaken the basal-type texture or form the basal pole split towards the TD by restricting the movement of grain boundaries, supporting the present result regarding the texture formed in the homogenized AXW100 alloy. The fine grain structure in the AXW100 sheet assures the improvement of the mechanical properties, especially, without scarifying the ductility.

## Conclusions

The tensile properties and microstructural evolution in relation to the age-hardenability and texture development of the AX10 and AXW100 alloy sheet have been investigated in this study. In the AXW100 alloy, the grain boundary motion is effectively retarded during the thermomechanical treatment by thermally stable Y-containing precipitates. Consequently, a relatively fine grain structure is maintained in the AXW100 alloy even after the homogenization treatment at 500 °C and a weak texture with the basal pole split towards the TD is formed, contrary to the coarse microstructure with a basal-type texture in the homogenized AX10 sheet. The peak-hardness of the AXW100 sheet is obtained after 1800 s of the aging treatment, accompanying the increase of YS from 162 to 244 MPa at the homogenized and peak-aged conditions, respectively. This improved YS is superior to the previously reported formable Mg sheet^1,12–14,17–19^ (see Table S1 in the supplementary material for the comparison). As a result, the alloying addition of Y into the AX10 alloy bring about highly beneficial effects, in terms of the improved YS and ductility at the same time arise from the fine microstructure by Y-containing thermally stable particles and significant aging response by the collaborative interaction of Al, Ca and Y solutes as well as texture weakening.

## Experimental procedure

 Mg–1Al–0.7Ca in wt% (AX10) and Mg–1Al–0.7Ca–0.7Y (AXW100) alloys were cast in an iron crucible under a protective atmosphere of Ar and SF_6_ mixture. After pouring the melt, the crucible was quenched in water. The chemical compositions measured by spark optical emission spectroscope (spark OES) were Mg–1.25Al–0.60Ca and Mg–0.94Al–0.68Ca–0.64Y for the AX10 and AXW100 alloys, respectively. The as-cast macrostructures of the AX10 and AXW100 alloys are shown in Fig. S1 in the supplementary material. The average grain size of the AX10 and AXW100 alloys are 810 $\upmu\text{m}$ and 1750 $\upmu\text{m}$, respectively. The rolling billets with a thickness of 10 mm were machined from the cast materials after the homogenization at 450 °C for 13 h, and then hot rolled at 450 °C to a final thickness of 1.2 mm with the step reduction degree of 10–20%. Intermediate annealing of the rolled sheet was conducted at 450 °C for 10 min prior to each rolling step. The rolled samples were solution treated at 500 °C for 3 h, and the aging treatments were performed at 250 °C up to 7200 s. Both heat treatments were conducted by using a fluidized sand furnace to ensure a homogeneous thermal distribution in the sample and precise annealing time. The hardness was measured using a Vickers hardness tester under a load of HV0.5 and was recorded as an average of 10 measurements at the longitudinal section of the aged sheet samples. Tensile tests were performed using the homogenized and peak-aged tensile samples with 40 mm in length and 18 mm in gauge length based on the DIN 50125, along the RD and TD at room temperature. The texture of the homogenized sheets was measured using a Panalytical X-ray diffractometer and Cu ${\text{K}}_{\alpha}$ radiation. The (0002) pole figures were recalculated using MTEX^45^. Specimens for TEM analysis were mechanically ground to a thickness of 100 µm then punched into a disk with 3 mm diameter. The disks were then electro-polished in a solution of 1.5% HClO_4_ with ethanol at − 30 °C, using a twin-jet polisher (Fischione, Model 110). TEM, STEM and EDS analysis were carried out using a JEOL JEM-F200 microscope.

## Supplementary Information

Supplementary Informations.

## References

[1] Bian MZ (2018). Bake-hardenable Mg–Al–Zn–Mn–Ca sheet alloy processed by twin-roll casting. Acta Mater..

[2] Kaneko J, Sugamata M, Numa M, Nishikawa Y, Takada H (2000). Effect of texture on the mechanical properties and formability of magnesium wrought materials. J. Jpn. Inst. Met..

[3] Basu I, Al-Samman T (2014). Triggering rare earth texture modification in magnesium alloys by addition of zinc and zirconium. Acta Mater..

[4] Mackenzie L, Pekguleryuz M (2008). The recrystallization and texture of magnesium–zinc–cerium alloys. Scr. Mater..

[5] Basu I, Al-Samman T, Gottstein G (2013). Shear band-related recrystallization and grain growth in two rolled magnesium-rare earth alloys. Mater. Sci. Eng. A.

[6] Zeng X (2019). Role of deformation mechanisms and grain growth in microstructure evolution during recrystallization of Mg–Nd based alloys. Scr. Mater..

[7] Kim YM (2017). Static recrystallization behaviour of cold rolled Mg––Y alloy and role of solute segregation in microstructure evolution. Scr. Mater..

[8] Zeng ZR (2016). Texture evolution during static recrystallization of cold-rolled magnesium alloys. Acta Mater..

[9] Hoseini-Athar MM, Mahmudi R, Prasath Babu R, Hedström P (2019). Effect of Zn addition on dynamic recrystallization behavior of Mg–2Gd alloy during high-temperature deformation. J. Alloys Compd..

[10] Zeng ZR (2019). Effects of calcium on strength and microstructural evolution of extruded alloys based on Mg–3Al–1Zn–0.3Mn. Metall. Mater. Trans. A.

[11] Suh B-C, Shim M-S, Shin KS, Kim NJ (2014). Current issues in magnesium sheet alloys: where do we go from here?. Scr. Mater..

[12] Nakata T (2020). New Mg–Al based alloy sheet with good room-temperature stretch formability and tensile properties. Scr. Mater..

[13] Chino Y, Huang X, Suzuki K, Mabuchi M (2010). Enhancement of stretch formability at room temperature by addition of Ca in Mg–Zn alloy. Mater. Trans..

[14] Shi R, Miao J, Luo AA (2019). A new magnesium sheet alloy and its multi-stage homogenization for simultaneously improved ductility and strength at room temperature. Scr. Mater..

[15] Bian, M. Z. *et al.* in *Magnesium Technology 2018 The Minerals, Metals & Materials Series* Ch. 55, 361–364 (2018).

[16] Bian MZ (2017). A heat-treatable Mg–Al–Ca–Mn–Zn sheet alloy with good room temperature formability. Scr. Mater..

[17] Pei R, Korte-Kerzel S, Al-Samman T (2019). Superior microstructure and mechanical properties of a next-generation AZX310 magnesium sheet alloy. Mater. Sci. Eng. A.

[18] Trang TTT (2018). Designing a magnesium alloy with high strength and high formability. Nat. Commun..

[19] Zhang Y (2019). Effects of alloying addition and twin-roll casting process on Mg–2Zn based-alloys with high strength and high formability. Mater. Res. Express.

[20] Takeuchi A, Inoue A (2005). Classification of bulk metallic glasses by atomic size difference, heat of mixing and period of constituent elements and its application to characterization of the main alloying element. Mater. Trans..

[21] Jayaraj J, Mendis CL, Ohkubo T, Oh-ishi K, Hono K (2010). Enhanced precipitation hardening of Mg–Ca alloy by Al addition. Scr. Mater..

[22] Suzuki A (2008). Precipitation strengthening of a Mg–Al–Ca-based AXJ530 die-cast alloy. Metall. Mater. Trans. A.

[23] Nakata T (2017). Strong and ductile age-hardening Mg–Al–Ca–Mn alloy that can be extruded as fast as aluminum alloys. Acta Mater..

[24] Mendis CL, Oh-Ishi K, Hono K (2012). Microalloying effect on the precipitation processes of Mg–Ca alloys. Metall. Mater. Trans. A.

[25] Oh-ishi K, Watanabe R, Mendis CL, Hono K (2009). Age-hardening response of Mg–0.3at.%Ca alloys with different Zn contents. Mater. Sci. Eng. A.

[26] Nie JF (2012). Precipitation and hardening in magnesium alloys. Metall. Mater. Trans. A.

[27] Nie JF, Muddle BC (1997). Precipitation hardening of Mg–Ca(–Zn) alloys. Scr. Mater..

[28] Oh J, Ohkubo T, Mukai T, Hono K (2005). TEM and 3DAP characterization of an age-hardened Mg–Ca–Zn alloy. Scr. Mater..

[29] Sandlöbes S (2012). The relation between ductility and stacking fault energies in Mg and Mg–Y alloys. Acta Mater..

[30] Nie JF, Muddle BC (2000). Characterisation of strengthening precipitate phases in a Mg–Y–Nd alloy. Acta Mater..

[31] Zhang M-X, Kelly PM (2003). Morphology and crystallography of Mg_24_Y_5_ precipitate in Mg–Y alloy. Scr. Mater..

[32] Sandlöbes S, Zaefferer S, Schestakow I, Yi S, Gonzalez-Martinez R (2011). On the role of non-basal deformation mechanisms for the ductility of Mg and Mg–Y alloys. Acta Mater..

[33] Bohlen J, Nürnberg MR, Senn JW, Letzig D, Agnew SR (2007). The texture and anisotropy of magnesium–zinc–rare earth alloy sheets. Acta Mater..

[34] Stanford N, Barnett M (2008). Effect of composition on the texture and deformation behaviour of wrought Mg alloys. Scr. Mater..

[35] Ha C (2019). Influence of Nd or Ca addition on the dislocation activity and texture changes of Mg–Zn alloy sheets under uniaxial tensile loading. Mater. Sci. Eng. A.

[36] Chino Y (2011). Effects of Ca on tensile properties and stretch formability at room temperature in Mg–Zn and Mg-Al alloys. Mater. Trans..

[37] Chen S-L (2002). The PANDAT software package and its applications. Calphad.

[38] Raghavan V (2010). Al–Ca–Mg (aluminum–calcium–magnesium). J. Phase Equilib. Differ..

[39] Suzuki A, Saddock N, Jones J, Pollock T (2004). Structure and transition of eutectic (Mg, Al)2Ca Laves phase in a die-cast Mg–Al–Ca base alloy. Scr. Mater..

[40] Dieter G, Bacon D (1986). Mechanical Metallurgy.

[41] Hidalgo-Manrique P, Robson JD, Pérez-Prado MT (2017). Precipitation strengthening and reversed yield stress asymmetry in Mg alloys containing rare-earth elements: a quantitative study. Acta Mater..

[42] Robson JD, Paa-Rai C (2015). The interaction of grain refinement and ageing in magnesium–zinc–zirconium (ZK) alloys. Acta Mater..

[43] Zarechnyuk OS, Drits ME, Rykhal RM, Kinzhibalo VV (1980). Study of a Magnesium–Aluminum–Yttrium system at 400 in the phase containing 0–33.3 at.% Yttrium. Izvestia Akademii nauk SSSR Metally.

[44] Zhou B-C, Shang S-L, Wang Y, Liu Z-K (2016). Diffusion coefficients of alloying elements in dilute Mg alloys: a comprehensive first-principles study. Acta Mater..

[45] Bachmann F, Hielscher R, Schaeben H (2010). Texture analysis with MTEX—free and open source software toolbox. Solid State Phenom..

