# Supplementary material for: Excellent age hardenability with the controllable microstructure of AXW100 magnesium sheet alloy
Source: Sci Rep. 2020 Dec 29;10:22413. doi: 10.1038/s41598-020-79390-z (PMC7772342; doi:10.1038/s41598-020-79390-z)
Supplement: Supplementary file 1 — Supplementary Informations. [file 41598_2020_79390_MOESM1_ESM.pdf]

Excellent age hardenability with the controllable microstructure of AXW100 magnesium sheet alloy

Sumi Jo*, Lawrence Whitmore, Sangkyu Woo, Ainhoa Urrutia Aramburu, Dietmar Letzig, Sangbong Yi*

*Corresponding author

Supplementary Material

S1. As-cast macrostructure of AX10 and AXW100 alloys

Fig. S1 shows the as-cast macrostructures of AX10 and AXW100 alloys. The macrostructure of AX10 and AXW100 alloys was not significantly changed after the homogenization treatment at 450 °C for 13 hrs conducted before rolling. The average grain sizes of the as-cast AX10 and AXW100 alloys are 810 μm and 1750 μm, respectively.

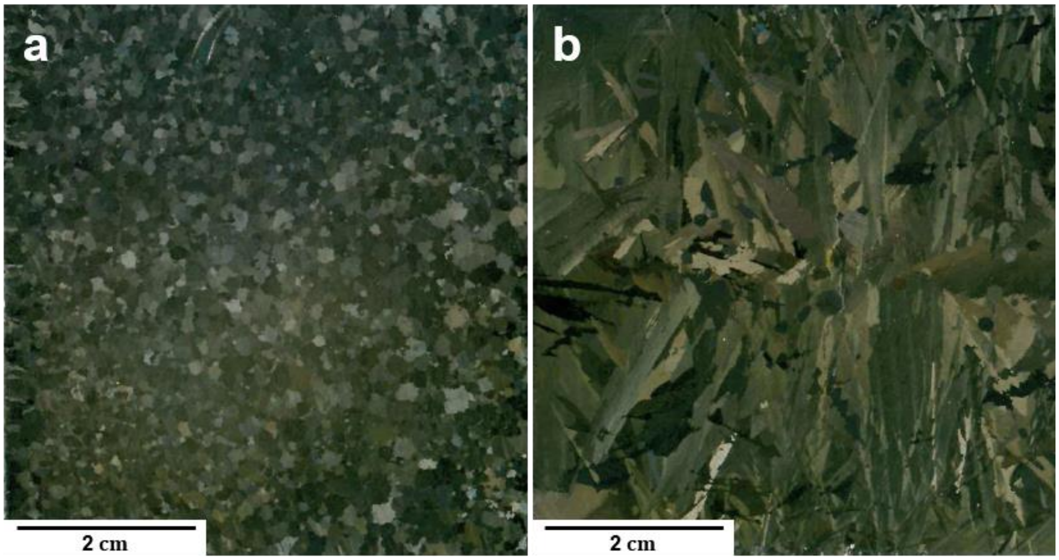

Fig. S1 As-cast macrostructures of AX10 and AXW100 alloys prior to rolling.

S2. Microstructure before and after aging treatment of AX10 sheet

Fig. S2 shows the TEM micrographs of the AX10 sheet before and after aging treatment. It is clear that a large amount of the fine precipitates, with an average size of 174 nm, are formed during the aging treatment and they lead to the precipitation hardening. The relatively large particles with an average size of 2.6 μm are the secondary phases inherited from the solidification process.

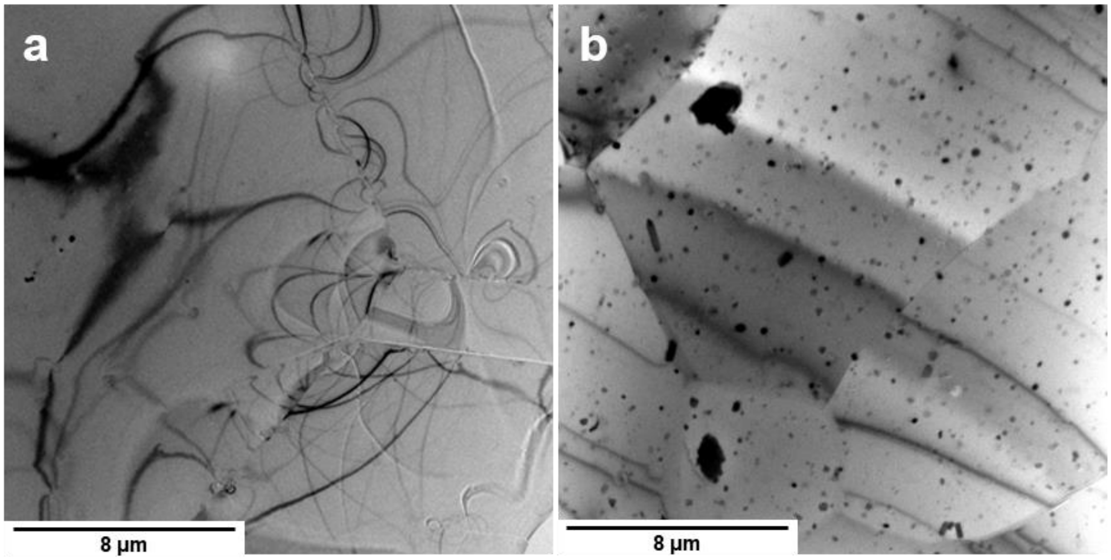

Fig. S2 TEM images of (a) the homogenized and (b) peak-aged AX10 sheet.

S3. Comparison of yield strength and room temperature ductility of the Mg sheets

Tab. S1 shows the comparison of yield strength and room temperature ductility of the Mg sheets including AXW100 alloy sheet investigated in this study. The AXW100 sheet shows the highest yield strength among the Mg alloy sheets in Tab. S1.

Alloys	Yield strength (MPa)	Formability or Ductility at RT	References
AZMW1110	238	26.3 %	[1]
AZ31	155	28 %	[12]
Mg-1.5Zn-0.1Ca	120	29 %	[13]
ZXEM2000	157	29 %	[14]
AZX310	190	30 %	[17]
AZMX3110	219	IE 8.0 mm	[18]
ZAEX2100	201	IE 6.23 mm	[19]
AXW100	244	18 %	present study

Tab. S1 Comparison of YS and room temperature ductility of Mg sheets.
